# Effect of curcumin compared to chlorhexidine on clinical variables of periodontal health: A systematic review and meta-analysis of randomized controlled trials

**DOI:** 10.1097/MD.0000000000049862

**Published:** 2026-07-24

**Authors:** Linxin Jiang, Simin Li, Daniel R. Reissmann, Gerhard Schmalz, Xianda Hu

**Affiliations:** a Department of Prosthodontics, University of Leipzig, Liebigstr. 12, Leipzig, Saxony, Germany; b Stomatological Hospital, School of Stomatology, Southern Medical University, Guangzhou, Guangdong, China; c Department of Conservative Dentistry and Periodontology, Brandenburg Medical School Theodor Fontane (MHB), Brandenburg an der Havel, Brandenburg, Germany; d China Tibetology Research Center, Laboratory of Molecular Cell Biology, Beijing Tibetan Hospital, Chaoyang, Beijing, China.

**Keywords:** chlorhexidine, curcumin, gingivitis, meta-analysis, periodontitis

## Abstract

**Background:**

Chlorhexidine is effective in managing periodontal diseases but has side effects. Curcumin is a natural alternative with anti-inflammatory properties. This meta-analysis aimed to compare the efficacy of curcumin and chlorhexidine on clinical variables in periodontal diseases.

**Methods:**

This study included only randomized controlled trials. The primary outcomes were differences in mean plaque index (PI) and gingival index (GI); the secondary outcomes were differences in probing depth (PD), attachment loss (AL), and bleeding index (BI). Data were analyzed by Stata software.

**Results:**

Twenty-six randomized controlled trials comprising 1266 subjects were included. Twenty-four studies had a high risk of bias, and 2 studies had some concerns. The pooled data revealed comparable efficacy of curcumin and chlorhexidine in reducing PI (*I*^2^ = 83.3%; standardized mean difference [SMD]: −0.041, 95% confidence interval [CI, −0.245–0.163], *P* = .692), GI (*I*^2^ = 87.8%; SMD: 0.033, 95% CI [−0.216–0.281], *P* = .797), and BI (*I*^2^ = 77.7%; SMD: −0.044, 95% CI [−0.208–0.295], *P* = .735). However, the efficacy of chlorhexidine was found to be superior to curcumin in reducing PD (*I*^2^ = 93.7%; SMD: 0.883, 95% CI [0.419–1.347], *P* < .001) and AL (*I*^2^ = 90.1%; SMD: 0.539, 95% CI [0.019–1.058], *P* = .042). As for the subgroup analyses of follow-up term, there was a nonsignificant difference between curcumin and chlorhexidine in PI, GI, and BI. As for PD and AL, the subgroup analyses of follow-up term showed different results from the overall pooled analysis: the short term (≤ 1 month) showed the superior efficacy of chlorhexidine over curcumin; however, the long term (> 1 month) showed similar efficacy.

**Conclusion:**

Curcumin could be an alternative to chlorhexidine and adjunctively used in reducing clinical variables of periodontal diseases, especially with regard to plaque accumulation and gingival inflammation.

## 1. Introduction

Periodontal disease, also known as periodontitis and/or gingivitis, is one of the most common inflammatory afflictions in oral disease.^[1]^ Gingivitis, the first stage of periodontal disease, is a reversible plaque-induced inflammation of the gingiva.^[2]^ When untreated, gingivitis may advance to periodontitis, characterized by inflammation of the surrounding and supporting tissues of the teeth, progressive loss of attachment, resorption of the alveolar bone, and tooth mobility.^[3]^ Mechanical plaque control via toothbrushing is the primary method to prevent and treat periodontal disease. Since mechanical plaque control is not practiced adequately by most individuals, adjunctive chemical plaque control must be used in order to eliminate dental plaque.^[4]^ Chlorhexidine, as a cationic bisbiguanide (1,6-bis [4-chloro-phenylbiguanido] hexane), has been declared the gold standard among all adjunctive chemical mouthwashes, mainly for its substantivity and broad-spectrum antibacterial activity.^[5]^ However, chlorhexidine is also reported to have various side effects, such as brown tooth staining, taste disturbance, and oral mucosal erosions, which limit its long-term application.^[6,7]^ Thus, to avoid or reduce these disadvantages of chlorhexidine, identification of alternative medicine is necessary.

Besides conventional medicine, traditional herbal medicines have been widely used for centuries, treating various oral health problems, including bleeding gums, halitosis, mouth ulcers, and tooth decay.^[8]^ Turmeric, also known as Haldi, the rhizome of the ginger family’s turmeric plant, has been widely used as a traditional Chinese herbal medicine in China and Asian countries such as India.^[9]^ As the active ingredient of turmeric, curcumin is characterized by its anti-inflammatory, antioxidant, antibacterial, antiseptic, and antimutagenic properties.^[10]^ Curcumin was suggested to exert anti-inflammatory effects by inhibiting the biological activity and synthesis of cyclooxygenase-2, lipoxygenase, and inducible nitric oxide synthase, which are essential proteases in mediating the inflammatory process.^[11]^ In addition, curcumin could suppress pro-inflammatory factors, such as leukotrienes, prostaglandins, and arachidonic acid, as well as neutrophil functions, thus acting as a potent anti-inflammatory during inflammation.^[11]^ In this context, many randomized controlled trials (RCTs) have been conducted to compare the efficacy of curcumin and commercially available chlorhexidine in the treatment and prevention of periodontal disease when applied in different ways (mouth rinses, local drug delivery gels, or chips).^[12–14]^ Recently, several systematic reviews and meta-analyses have been conducted to assess the efficacy of curcumin and chlorhexidine in treating gingivitis and periodontitis. However, these analyses have incorporated few or no large-sample studies (≥ 50 participants), thus limiting the generalizability of their findings.^[15–18]^

This study aims to comprehensively analyze and compare the clinical efficacy of curcumin and chlorhexidine in patients with periodontal disease, including gingivitis and periodontitis. To enhance the generalizability and reliability of the findings, RCTs were included, with a focus on studies involving large-sample sizes. Through meta-analysis, this study seeks to provide more comprehensive and clinically relevant evidence to evaluate the feasibility of curcumin in periodontal disease management and explore its potential as an alternative treatment to chlorhexidine. Furthermore, the following hypotheses were proposed: curcumin is not inferior to chlorhexidine in reducing plaque accumulation and gingival inflammation; the efficacy differences between curcumin and chlorhexidine are influenced by the duration of follow-up.

## 2. Materials and methods

### 2.1. Participants-interventions-comparisons-outcomes study design question

This systematic review was performed to answer the focused question: “How are the effects of curcumin for plaque and inflammation control as a supplement to daily oral hygiene compare to chlorhexidine among patients with periodontal diseases?” according to the following elements:

(1) Participants (P): systematically healthy participants with periodontal diseases;
(2) Interventions (I): the application of curcumin products using different application methods, for example, general full-mouth method (mouthwash), and topical subgingivally used methods (topical gel, chip, irrigation solution, syringe);
(3) Comparisons (C): the application of chlorhexidine products using different application methods, for example, general full-mouth method (mouthwash), and topical subgingivally used methods (topical gel, chip, irrigation solution, syringe);
(4) Outcomes (O): the clinical effects of mouthwashes as a supplement to daily oral hygiene (i.e., toothbrushing) on the control of periodontal inflammation, which was manifested by the decrease of these following parameters: plaque index (PI), gingival index (GI), probing depth (PD), attachment loss (AL), and bleeding index (BI).
(5) Study design (S): RCTs only.

### 2.2. Search strategy

A systematic search of PubMed, EMBASE, and the Cochrane Library Register of Controlled Trials online databases was performed, covering studies from inception to January 29, 2025, for all RCTs that compared curcumin with chlorhexidine in treating periodontal diseases and reported the periodontal-related short-term and long-term outcomes. The detailed searching strategies, aligned with this time frame, for 3 databases were respectively listed in Tables S1 to S3, Supplemental Digital Content 1, and the summarized searching strategy was listed in Table S4, Supplemental Digital Content 4. In addition, manual searches for references cited in the published original and review articles were also performed. The language of publications was restricted to English only. Unpublished works were not accounted for. The searching flowchart was plotted according to the preferred reporting items for systematic review and meta-analysis flow diagram, especially for depicting the flow of information through the different phases of a systematic review.^[19]^ The flow diagram maps out the number of records identified, included, and excluded, and the reasons for exclusions. The template named as “preferred reporting items for systematic review and meta-analysis 2020 flow diagram for new systematic reviews which included searches of databases, registers and other sources” was used in this research (uniform resource locator: https://www.prisma-statement.org/prisma-2020-flow-diagram).

### 2.3. Eligibility criteria

The studies that involved human participation were included only if they met certain criteria, which are: the type of study design must be RCT; the patient involved is clinically diagnosed as having gingivitis or periodontitis, and also without suffering from any chronic systemic inflammatory diseases; controlled randomized design with at least 2 treatment arms, of which 1 must be related to the use of curcumin, and the other must be related to the use of chlorhexidine; changes in at least 1 of the periodontal disease-related outcomes PI, GI, PD, AL, BI were recorded in the study; there is a mean follow-up period of at least 1 week.

The exclusion criteria for our study were: observational studies (e.g., cohort, cross-sectional, or case-control), systematic reviews and meta-analyses, case reports and case series, as well as letters to the editor, editorials, reviews, and commentaries; abstracts from conference proceedings, because their results are often not final, and they contain insufficient information to thoroughly assess study quality); studies performed on cultured cells or animal models; potential participants who had chronic systemic inflammatory diseases; studies lacked Chlorhexidine as the control group; studies that didn’t record any of the periodontal disease-related parameters.

### 2.4. Data extraction

The data extracted from each article by 2 investigators (L.J. and S.L.) included the following: first author, year of publication, publication period (recently within 5 years/earlier studies of 5 years prior), disease type of subjects (i.e., whether gingivitis or periodontitis), SRP (scaling and root planning) treatment (i.e., having received or not received), type of drug application (i.e., general full-mouth [e.g., mouthwash], or topical application [e.g., subgingival irrigation solution, subgingival gel/chip, gel in teeth shield, and photodynamic antimicrobial chemotherapy using LED light]), timepoint of follow-up (i.e., short term within 1 month, or long term of longer than 1 month), age of participants (i.e., whether adolescents or adults), sample size of included RCTs (small sample size [n < 50], or larger sample size [n ≥ 50]), changes in 4 outcomes (PI, GI, AL, and BI), and the pharmaceutical formulation, vehicle, and concentration of curcumin and chlorhexidine used in each trial (e.g., aqueous mouthrinse, subgingival gel or chip, irrigation solution, or nanocarrier-based/ self-nanoemulsifying delivery system). Data were extracted at baseline and at follow-up for the clinical parameters (Table S2, Supplemental Digital Content 2). A third researcher addressed all remaining discrepancies after consultation between the 2 investigators.

### 2.5. Quality assessment

The quality of RCTs was considered and assessed according to version 2 of the risk-of-bias (RoB) tool for randomized trials, which is a revised Cochrane RoB tool for randomized trials.^[20]^ Version 2 of the RoB tool for randomized trials is provided by the Cochrane Handbook for Systematic Reviews of Intervention version 7 (updated March 2021).^[21]^ RoB 2 will be used to evaluate the RoB in each included study by 2 independent authors (i.e., SL and LJ). The main items include 5 domains, for example: randomization process (random sequence generation, allocation sequence concealment, difference between intervention groups); timing of identification or recruitment of participants; deviation from intended interventions; missing outcome data; measurement of the outcome; and selection of the reported result. According to the overall RoB judgment, each bias is divided into 3 levels: including low risk, high risk, and some concerns. If there are any differences in the evaluation, they will be discussed and negotiated with the third author to reach a consensus.

### 2.6. Statistical analysis

All of the statistical analyses were conducted using Stata (the newest version 18.0 released on April 2023, Stata Corp). The descriptive statistics were presented as mean ± standard error. The differences (experimental [curcumin] minus control [chlorhexidine]) of the changes (final values minus baseline values) were employed to calculate the net changes of PI, GI, PD, AL, and BI between the 2 groups. The standardized mean difference before and after intervention with 95% confidence intervals (CIs) was calculated as a measure of treatment effect. The calculation was performed based on the method outlined in the Cochrane Handbook for Systematic Reviews of Interventions version 6.5 (updated August 2024) (https://www.cochrane.org/handbook).

Afterward, the Cochran *Q* test and *I*^2^ statistics were used to estimate the statistical heterogeneity among the included studies. The type of model used depended on the *Q* test results. *P* < .05 or *I*^2^ > 50% represented a substantially high level of heterogeneity. A random-effect model would be used if there was considerable heterogeneity as shown by *P* < .05 (*Q* test) or *I*^2^ >50%. Otherwise, a fixed-effect model would be used if there was a low level of heterogeneity as shown by *P* > .05 (*Q* test) and *I*^2^ < 50%. The methodology in this systematic review was based on the guidelines in the Meta-Analysis Report Quality statement.^[22]^

Subgroup analyses were conducted according to several covariables including published time, disease type, SRP treatment, application type, follow-up time, age of subjects, and sample size. Meta-regression analysis was also carried out by fitting the covariables one-by-one into a meta-regression model. If there was a suggested association, the subsequent sensitivity analysis excluding the outlier studies and/or a subgroup analysis was performed.

If there is difficulty in detecting underlying heterogeneity in small meta-analyses and inaccurate estimates, sensitivity analyses using a range of heterogeneity levels would be performed.^[23]^ Sensitivity analysis was conducted to explore, quantify, and control for sources of heterogeneity and stability of results across studies by excluding eligible studies by sequence.

Publication bias for each outcome of interest was also investigated by visual assessment in the funnel plot^[24]^ and the quantitative Egger test.^[25]^ Egger test was employed to identify the statistical significance of publication bias.^[25]^

## 3. Results

### 3.1. Searching flowchart

**Figure 1.**
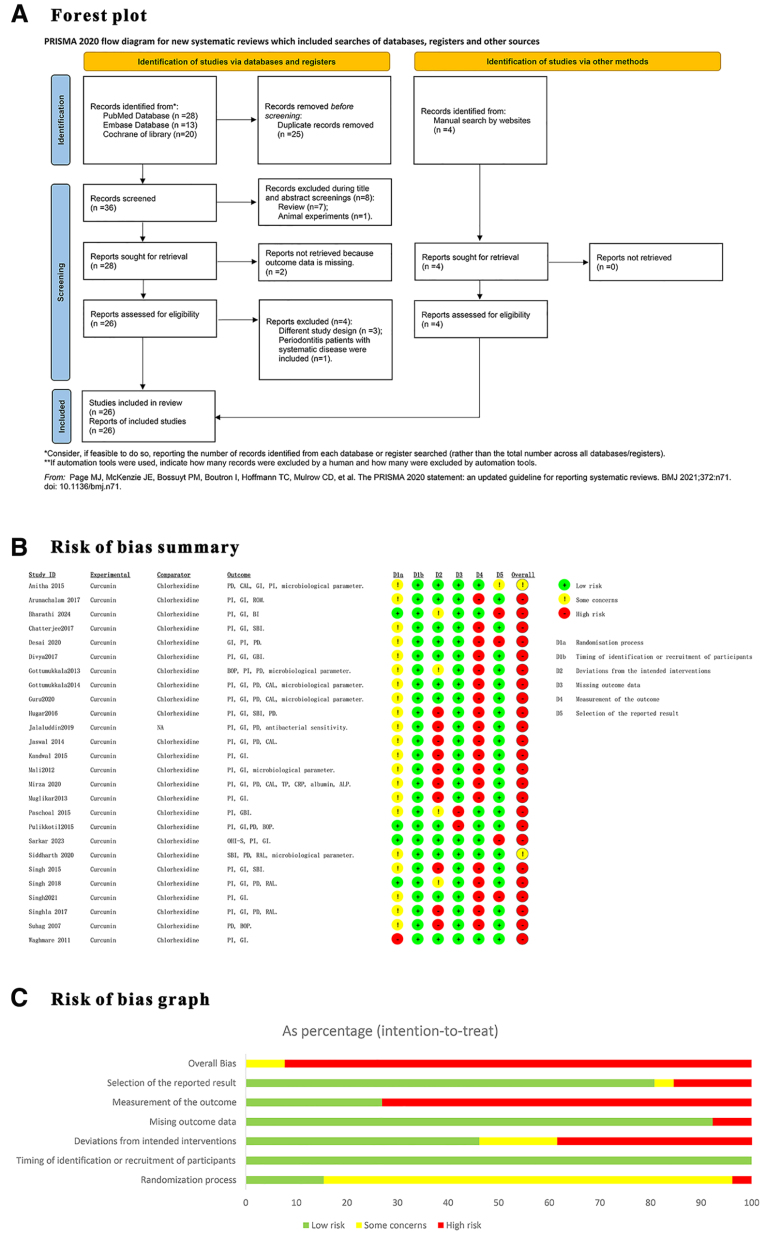
The searching flowchart to obtain RCTs and the quality evaluation of these studies. (A) The PRISMA 2020 flow diagram for new systematic reviews which included searches of databases, registers and other sources; (B) the RoB summary; (C) the RoB graph. RCT = randomized controlled trial, RoB = risk-of-bias, n = number of records, PRISMA = preferred reporting items for systematic review and meta-analysis.

As shown in Figures 1A, 61 papers were accessed from the 3 electronic databases, with 25 duplicates removed. In the remaining 36 papers, 8 papers were excluded during title and abstract screenings, 2 papers were excluded for missing outcome data, 3 papers were excluded for a different study design, and 1 paper was excluded for including periodontitis patients with systematic disease. Additionally, 4 papers were accessed from the manual search for references cited in the published original and review article.

Thus, 26 relevant RCTs with 1266 participants were finally included in the analysis of the current systematic review.

### 3.2. RoB assessment

The RoB summary (Fig. 1B) and RoB graph (Fig. 1C) were presented respectively. 24 studies were evaluated as having a high RoB, while 2 studies were judged as having some concerns (Anitha 2015, and Siddharth 2020).^[26,27]^ Table S5, Supplemental Digital Content 5 shows the RoB assessment results for each included study.

### 3.3. Descriptive analysis

The characteristics of the 26 studies are presented in Table S6, Supplemental Digital Content 6. Eighteen studies were published before 2019 (5 years ago) while the remaining 8 studies were published recently, on and after 2019 (within the recent 5 years). The age of included subjects also varied: 1 study^[28]^ included subjects aged 13 to 18, 2 studies^[29,30]^ included subjects older than 15, and the remaining 23 studies included subjects older than 18 years. The sample size of the studies varied: the sample size of 6 studies was more than 50, while the sample size of the other 20 studies was < 50. Fourteen studies included gingivitis patients, and the remaining 11 studies included periodontitis patients. Ten studies reported the long term (> 1 month), while the remaining 16 studies reported the short term (≤ 1 month). Regarding whether the included subjects had received SRP treatment, the subjects of 9 studies didn’t receive the SRP treatment, and the subjects of the remaining 17 studies had received the SRP treatment. In terms of the application type of the drugs, these studies varied: 8 studies applied a general full-mouth mouthwash, and the remaining 18 studies applied the topical way to be the treatment.

### 3.4. Meta-analysis results of primary outcomes

**Figure 2.**
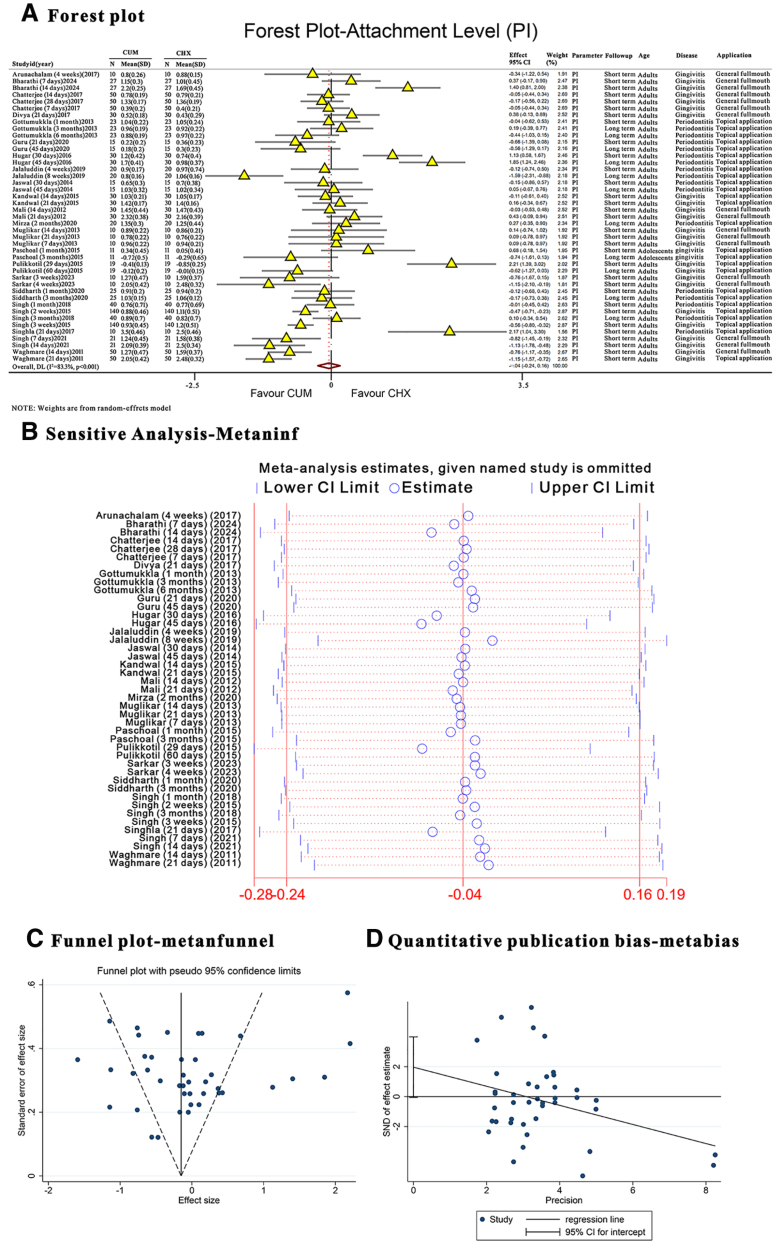
Meta-analysis results for the primary outcome: PI. (A) Forest plot for PI outcome; (B) sensitivity analysis results for PI outcome; (C) funnel plot for PI outcome; (D) quantitative publication bias results for PI outcome. CHX = chlorhexidine, CI = confidence interval, CUM = curcumin, PI = plaque index, SD = standard deviation, SND = standard normal deviate.

**Figure 3.**
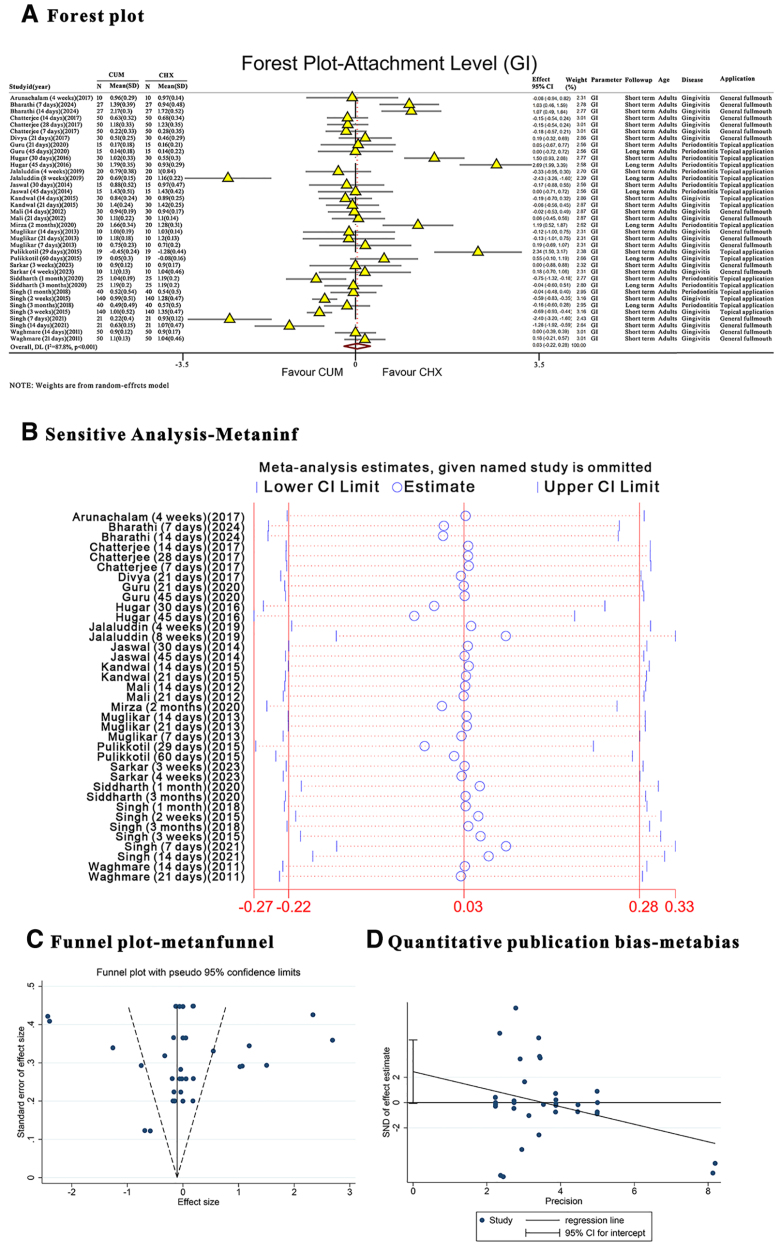
Meta-analysis results for the primary outcome: GI. (A) Forest plot for GI outcome; (B) sensitivity analysis results for GI outcome; (C) funnel plot for GI outcome; (D) quantitative publication bias results for GI outcome. CHX = chlorhexidine, CI = confidence interval, CUM = curcumin, GI = gingival index, SD = standard deviation, SND = standard normal deviate.

Twenty-two RCTs (43 tests) met the criteria examining the PI outcome (Fig. 2A), and 19 RCTs (37 tests) met the criteria examining the GI outcome (Fig. 3A). For both PI and GI outcomes, the differences between curcumin and chlorhexidine were not statistically significant, with large heterogeneity.

### 3.5. Meta-analysis results of secondary outcomes

**Figure 4.**
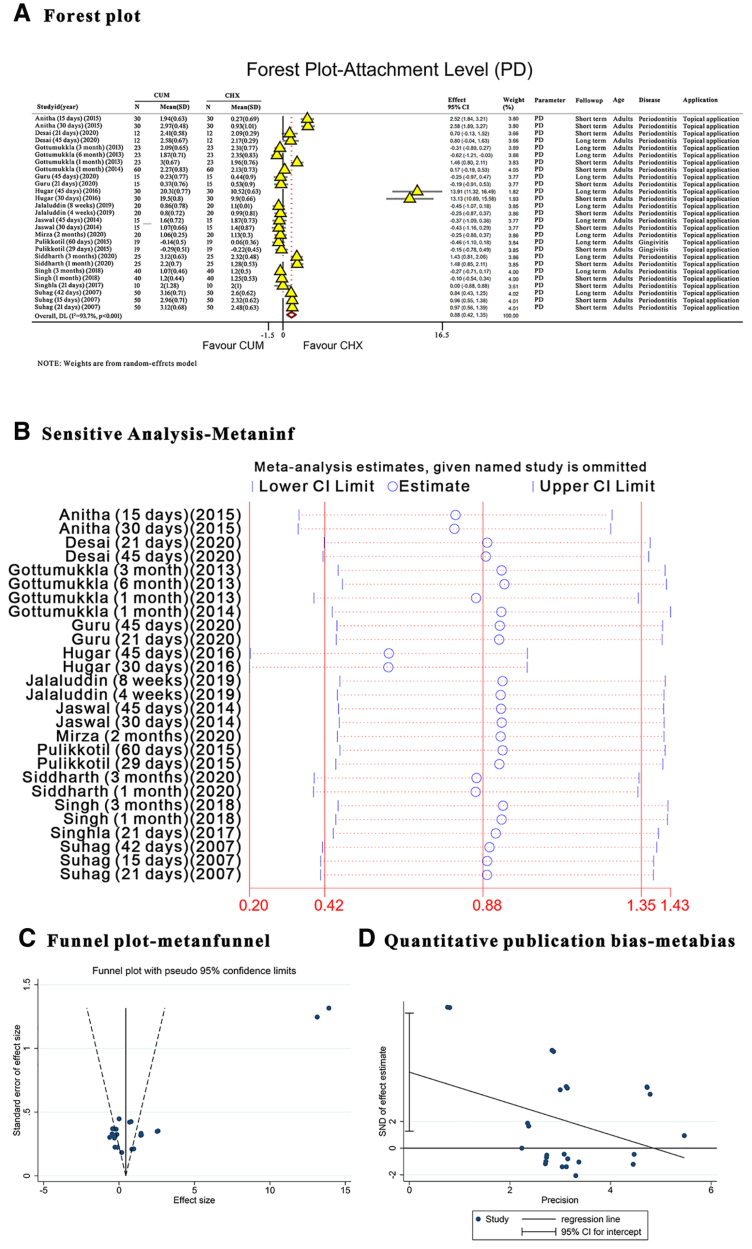
Meta-analysis results for the secondary outcome: PD. (A) Forest plot for PD outcome; (B) sensitivity analysis results for PD outcome; (C) funnel plot for PD outcome; (D) quantitative publication bias results for PD outcome. CHX = chlorhexidine, CI = confidence interval, CUM = curcumin, PD = probing depth, SD = standard deviation, SND = standard normal deviate.

**Figure 5.**
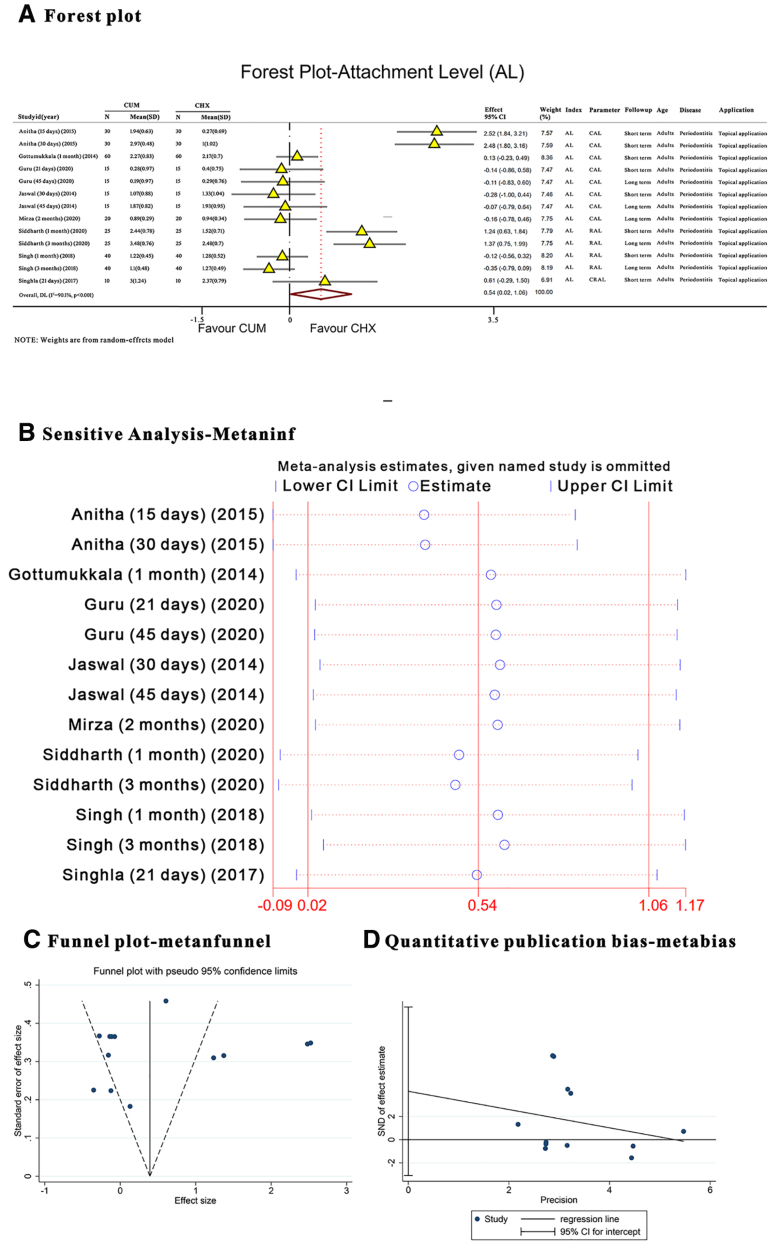
Meta-analysis results for the secondary outcome: AL. (A) Forest plot for AL outcome; (B) sensitivity analysis results for AL outcome; (C) funnel plot for AL outcome; (D) quantitative publication bias results for AL outcome. AL = attachment loss, CHX = chlorhexidine, CI = confidence interval, CUM = curcumin, SD = standard deviation, SND = standard normal deviate.

**Figure 6.**
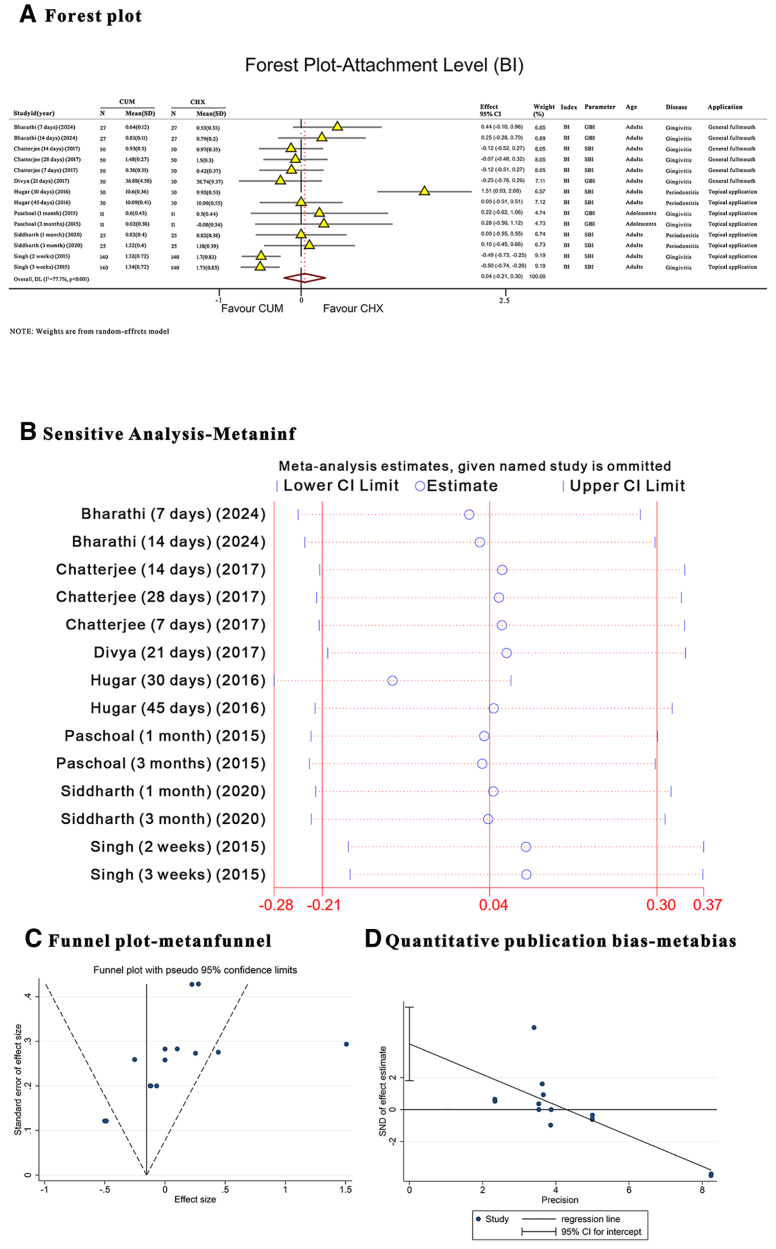
Meta-analysis results for the secondary outcome: BI. (A) Forest plot for BI outcome; (B) sensitivity analysis results for BI outcome; (C) funnel plot for BI outcome; (D) quantitative publication bias results for BI outcome. BI = bleeding index, CHX = chlorhexidine, CI = confidence interval, CUM = curcumin, SD = standard deviation, SND = standard normal deviate.

Fourteen RCTs (27 tests) met the criteria examining the PD outcome (Fig. 4A), and 8 RCTs (13 tests) met the criteria examining the AL outcome (Fig. 5A). PD and AL reduction after applying chlorhexidine was greater than after applying curcumin, with large heterogeneity. Seven RCTs (14 tests) met the criteria examining the BI outcome (Fig. 6A), and the differences between curcumin and chlorhexidine were not statistically significant, with large heterogeneity.

### 3.6. Results of sensitivity analysis

The sensitivity analyses for 5 outcomes were respectively performed by omitting 1 study at a time to gauge the robustness of the results. As for PI (Fig. 2B), GI (Fig. 3B), PD (Fig. 4B), and BI (Fig. 6B), the pooled effect was not significantly altered by excluding any single study. None of the individual studies influenced the pooled effects. This indicated that the meta-analysis results were statistically robust.

AL reduction after applying chlorhexidine was greater than after applying curcumin (standardized mean difference = 0.539, 95% CI [0.019–1.058], *P* = .042). However, after omitting tests from Anitha 2015, Gottumukkala 2014, Siddharth 2020, or Singhla 2017, the differences between curcumin and chlorhexidine were no longer statistically significant. Thus, the meta-analysis results regarding AL were not statistically robust, reliable, or stable (Fig. 5B).

### 3.7. Results of subgroup analysis

In order to identify the contributing factors underlying heterogeneity, subgroup analyses by publication time, disease type, SRP treatment, application type, follow-up time, age of subjects, and sample size were conducted, respectively, for 5 different outcomes (Table S7, Supplemental Digital Content 7).

As for PI and GI, the subgroup analyses based on all of the investigated covariates obtained similar results as the overall pooled effects, showing similar effects between curcumin and chlorhexidine. As for PD, subgroup analyses based on recent studies within 5 years, subjects with gingivitis, subjects without having received SRP treatment, and follow-up time longer than 1 month showed similar effects between curcumin and chlorhexidine. The overall and other subgroup analyses showed the better effects of chlorhexidine over curcumin. As for AL, subgroup analyses based on publication time, follow-up time longer than 1 month, and sample size showed similar effects between curcumin and chlorhexidine. As for BI, except for the subgroup analyses of the subjects without receiving SRP treatment, all of the covariates obtained similar results as the overall pooled effects, showing similar effects between curcumin and chlorhexidine.

### 3.8. Results of meta-regression analysis

Random-effect meta-regression analyses were used to explore the possible sources of heterogeneity among the studies (Table S8, Supplemental Digital Content 8). All the investigated moderators had no significant influence on study outcome.

### 3.9. Results of publication bias

Each of the comparisons included more than 10 tests, which met the minimum requirement for an analysis using a funnel plot.

The funnel plots of the effect of PI (Fig. 2C), GI (Fig. 3C), and AL (Fig. 5C) reduction when comparing curcumin and chlorhexidine appear to be generally symmetric. The results from the Egger tests (Fig. 2D, Fig. 3D, Fig. 5D) also indicated nonsignificant publication bias (Egger test: *P* > .05). The test of H0 shows that there were no small-study effects in this analysis.

The funnel plots of the effect of PD (Fig. 4C) and BI (Fig. 6C) reduction when comparing curcumin and chlorhexidine appear to be apparently nonsymmetric. The results from the Egger tests (Fig. 4D, Fig. 6D) also indicated significant publication bias (Egger test: *P* > .05). And test of H0 shows that there were small-study effects in this analysis.

## 4. Discussion

The results obtained from this meta-analysis showed that the efficacy of curcumin in reducing PI, GI, and BI was comparable to that of chlorhexidine. Regarding the reduction of periodontitis-specific parameters (PD and AL) chlorhexidine shows superior effects over curcumin in the short term, while both drugs show similar effects in the long term. The comparable effects of both drugs in adjunctively treating periodontal diseases should be due to the antibacterial, anti-inflammatory, antioxidant, anti-collagenase, and anti-osteoclastogenesis properties, which have been well demonstrated to be the pharmacological effects of both drugs. Given its favorable systemic safety profile, curcumin may be considered as a potential alternative or adjunct to chlorhexidine in the adjunctive management of periodontal diseases; however, its cost advantage should not be overstated, as it depends on the required purity and the formulation used.

The antibacterial mechanisms of the 2 drugs in combating periodontal inflammation vary. Chlorhexidine plays an anti-bactericidal role in disrupting the cellular membrane’s permeability, as the positively charged chlorhexidine molecules could bind the negatively charged bacteria cell walls. The disruption of osmotic equilibrium causes the coagulation of intracellular cytoplasmic macromolecules and, ultimately, cellular death.^[31]^ Due to this mechanism, apart from destroying the pathogenic bacteria, chlorhexidine could also kill the normal commensal bacteria that might protect against infection. In addition, chlorhexidine also binds to the acidic groups of salivary acidic glycoproteins and seals the acidic groups of salivary glycoproteins. It could reduce the capability of salivary glycoproteins in adsorbing to the tooth surface and inhibit the formation of acquired biofilms and plaque.^[32]^ Contrary to the mechanisms of chlorhexidine, curcumin exerts its antibacterial mechanism by damaging the DNA in the bacterial cells, which further alters the chromosomes’ structure.^[33]^ Despite previous evidence on curcumin’s antibacterial role against various oral pathogens (e.g., *Prevotella intermedia*, *Porphyromonas gingivalis*, *Streptococcus mutans*), further in vitro microbiological studies are needed to investigate the detailed underlying mechanism of curcumin.^[34–36]^

It should be noted that curcumin is highly lipophilic and poorly soluble in water, which markedly limits its bioavailability when delivered as a simple aqueous preparation. To overcome this limitation, the included trials employed a variety of formulations and delivery vehicles rather than a single standardized product, ranging from aqueous mouthrinses prepared from turmeric/curcumin extract to locally delivered subgingival gels and chips, irrigation solutions, and bioavailability-enhanced systems such as nanocarrier-based or self-nanoemulsifying curcumin. The concentration of curcumin also differed substantially between studies. Because the formulation and vehicle directly influence the local availability and substantivity of curcumin, this heterogeneity in delivery systems is likely to be an important source of the high between-study heterogeneity observed in the present meta-analysis and should be taken into account when interpreting the pooled estimates.

Apart from antimicrobial effects, curcumin exerts anti-inflammatory, antioxidant, and anti-collagenase properties, which are also possessed by chlorhexidine. The 2 drugs both dramatically reduced the GI and PI in periodontal diseases. Curcumin can suppress the metabolism of arachidonic acid, playing a similar anti-inflammatory role to aspirin. The anti-inflammatory effects of curcumin manifested in 2 aspects: on 1 hand, curcumin inhibited the synthesis of prostaglandin-2 and thromboxane without altering prostacyclin levels; on the other hand, curcumin decreases the expression of various inflammatory mediators, like interleukin (IL)-1β, IL-8, IL-6, and tumor necrosis factor-α, via mediating transcription factors and signaling pathways like nuclear factor-kB, activator protein-1, and mitogen-activated protein kinase.^[37,38]^ Likewise, chlorhexidine can suppress the production of pro-inflammatory cytokines by inhibiting the migration of oral polymorphonuclear leukocytes.^[39,40]^

Antioxidant mechanisms can be implicated as an indirect manifestation of antimicrobial mechanisms. Cell death is controlled by several intracellular factors, including the disruption of a stable redox balance that develops between reactive oxygen species and the antioxidant defense system.^[41]^ It has been shown that a positive correlation exists between oxidative stress and the severity of periodontal disease.^[42]^ Curcumin and chlorhexidine both possessed antioxidant and radioprotective ability, protecting cells against oxidative stress-induced damage, thus acting as therapeutic agents in preventing and treating periodontal disease. The antioxidant activity of curcumin is suggested to be equivalent to Vitamin C and Vitamin E, which has been confirmed to scavenge a variety of reactive oxygen species, including superoxide anion radicals, hydroxyl radicals, and nitrogen dioxide radicals.^[43,44]^ Chlorhexidine could induce cell apoptosis by promoting ROS production and facilitating the use of reactive oxygen species as a pro-oxidant.^[45]^

BI is regarded as a vital marker of wound healing. The forest plot results obtained in the current meta-analysis showed the similar effects of both drugs in reducing BI. This is not within our expectation based on the results from many previous literature reporting the delayed wound healing effects of chlorhexidine. Chlorhexidine, as an antiseptic, can delay wound healing by inhibiting the functions of fibroblasts in the healing processes.^[46]^ However, curcumin can promote the migration of fibroblasts to the wound sites and increase the transcription of fibronectin and transforming growth factor-beta, thus accelerating wound healing.^[47]^ Curcumin could significantly reduce BI by reducing inflammatory edema and vasodilatation in the collared hoof tissue, as well as suppressing angiogenesis through fibrosis of collared hoof tissue.^[48]^

In addition, curcumin has an obvious advantage over chlorhexidine in terms of systematic safety. Curcumin has been prescribed for systemic use by Ayurvedic, Tibetan, and many other traditional medical practitioners to treat a host of illnesses for thousands of years. It has shown a definite curative effect without apparent adverse effects. Theoretically, even if patients swallow curcumin mouthwash, there will be hardly any harmful impact. In contrast, the ingestion of chlorhexidine brings systemic toxicity, which might need emergent treatment. For instance, if patients accidentally swallow chlorhexidine products, they may suffer headaches, giddiness, mild mist, euphoria, stomachache, and diarrhea. Furthermore, curcumin also has advantages in taste, no staining of teeth, and low price. When comparing taste between both medicines, curcumin tastes only a little bitter, while chlorhexidine has a lingering taste and primarily causes taste confusion.^[5]^ Regarding production cost, although turmeric (*Curcuma longa*) itself is a widely available and inexpensive raw material, pure curcumin accounts for only approximately 1 to 3% of the dried rhizome, and obtaining pharmaceutical-grade curcumin requires an elaborate process involving solvent (e.g., acetone) extraction of the oleoresin, complete evaporation of the solvent, and subsequent crystallization. The cost of curcumin relative to chlorhexidine therefore varies considerably with the required purity and formulation, and should not be assumed to be uniformly lower. According to a survey by the World Health Organization, up to 4 billion people (representing 80% of the world’s population) living in developing countries rely on herbal medicinal products as a primary source of the healthcare system.^[49]^ However, it must be emphasized that the safe and effective use of any medication depends on its administration at a verified, standardized strength. Because the potency of curcumin preparations varies markedly with the source material, extraction method, and formulation, non-standardized curcumin products cannot be recommended for use outside a controlled clinical or pharmacy setting on the basis of the present evidence. Standardized, quality-controlled curcumin formulations would therefore be required before curcumin could be regarded as a reliable alternative to commercially available chlorhexidine in any population, including in rural or low-resource settings.

The PD and AL are the most commonly used clinical measures in epidemiological studies for periodontitis. These 2 parameters are also utilized as a minimum diagnostic threshold for defining periodontitis at a given site, in terms of an AL of 3mm and a PD of 3 mm. Greater PD and AL reflect increased alveolar bone loss and the severity and progression of periodontitis. The current findings show the superior efficacy of chlorhexidine over curcumin in reducing PD and AL within the short term of follow-up, while showing similar efficacy of both drugs in reducing PD and AL within the long term of follow-up. The mediating role of both drugs in bone resorption activity has been investigated by many previous studies. Another recent investigation showed that using chlorhexidine gel inside the fixture/abutment of a dental implant connection can reduce peri-implant marginal bone loss.^[50]^ Although the inhibiting role of chlorhexidine in bone loss has been well demonstrated, a recent study established an experimental periodontitis model in rats and found the inhibiting role of curcumin in bone destruction by the underlying mechanisms of regulating RANKL and IL-1β markers.^[51]^ Although both drugs are capable of preventing bone resorption, their exact mechanisms of action in impairing osteoclastic activity remain unclear. The current finding, summarized by clinical trials, therefore needs to be validated by designing in vitro cellular experiments to observe the influence of both drugs on osteoclastic activity and further investigate the underlying mechanisms.

## 5. Limitations

Despite the strict inclusion and exclusion criteria and the rigorous quality evaluation of this meta-analysis, the following limitations of this study existed. First, the quality of the majority of RCTs showed a high RoB, and there was a lack of high-quality RCTs with rigorous methods of random sequence generation and a double-blind strategy. Most of the included studies did not describe specific random allocation methods, blinding, and implementation of allocation concealment schemes. Only 6 studies used blinding, and these factors led to bias in the included studies. Second, most of the studies included had small sample sizes of < 50. Furthermore, the majority of RCTs generally lasted < 1 month. Given the limitations of this meta-analysis, further trials with longer terms of follow-up and more comprehensive outcomes are expected to better compare these 2 medicines’ efficacy in combating periodontal diseases. Third, the included trials used heterogeneous curcumin formulations, vehicles, and concentrations (from aqueous mouthrinses to nanocarrier-based gels and chips); because the intrinsic water solubility of curcumin is poor, its local bioavailability is strongly formulation-dependent, and this variability could not be fully accounted for and and may partly explain the substantial heterogeneity observed across studies.

## 6. Conclusion

Available evidence suggests that curcumin has equivalent effects to chlorhexidine in reducing plaque accumulation and gingival inflammation. Chlorhexidine presents superior effects over curcumin in inhibiting alveolar bone loss in the short term; however, both drugs obtained similar effects in the long term. Therefore, curcumin could be viewed as an alternative medicine to chlorhexidine to be used as an adjuvant treatment for periodontal diseases. However, considering the limitations in the included studies, the conclusion should be interpreted with caution. Additional high-quality studies are warranted to compare the anti-gingivitis and anti-periodontitis effects of curcumin and chlorhexidine in the longer term.

## Author contributions

**Conceptualization:** Simin Li, Xianda Hu.

**Data curation:** Daniel R. Reissmann.

**Formal analysis:** Linxin Jiang.

**Funding acquisition:** Linxin Jiang, Simin Li.

**Methodology:** Daniel R. Reissmann.

**Project administration:** Gerhard Schmalz.

**Resources:** Gerhard Schmalz.

**Software:** Linxin Jiang.

**Supervision:** Gerhard Schmalz, Xianda Hu.

**Validation:** Daniel R. Reissmann.

**Visualization:** Linxin Jiang.

**Writing – original draft:** Linxin Jiang.

**Writing – review & editing:** Daniel R. Reissmann, Gerhard Schmalz, Xianda Hu.
















