# Supplementary material for: Effect of curcumin compared to chlorhexidine on clinical variables of periodontal health: A systematic review and meta-analysis of randomized controlled trials
Source: Medicine (Baltimore). 2026 Jul 24;105(30):e49862. doi: 10.1097/MD.0000000000049862 (PMC13406067; doi:10.1097/MD.0000000000049862)
Supplement: Supplementary file 1 [file medi-105-e49862-s001.docx]

**Supplementary Table 1**

The detailed searching strategy in PubMed database. Date of search: January 29, 2025.

| **Searching steps** | **Searching strategy of each step** |
| --- | --- |
| **#1** | (periodontal diseases[MeSH Terms]) OR (((((periodontitis[Title/Abstract]) OR (gingivitis[Title/Abstract])) OR (gum disease[Title/Abstract])) OR (gingival inflammation[Title/Abstract])) OR (chronic periodontitis[Title/Abstract])) |
| **#2** | (curcumin[MeSH Terms]) OR (((curcumin[Title/Abstract]) OR (curcuma[Title/Abstract])) OR (turmeric[Title/Abstract])) |
| **#3** | (chlorhexidine[MeSH Terms]) OR (chlorhexidine[Title/Abstract]) |
| **#4** | #1 and #2 and #3  (((periodontal diseases[MeSH Terms]) OR (((((periodontitis[Title/Abstract]) OR (gingivitis[Title/Abstract])) OR (gum disease[Title/Abstract])) OR (gingival inflammation[Title/Abstract])) OR (chronic periodontitis[Title/Abstract]))) AND ((curcumin[MeSH Terms]) OR (((curcumin[Title/Abstract]) OR (curcuma[Title/Abstract])) OR (turmeric[Title/Abstract])))) AND ((chlorhexidine[MeSH Terms]) OR (chlorhexidine[Title/Abstract])) |
| **#5** | ((((randomized controlled trial[Publication Type]) OR (controlled clinical trial[Publication Type])) OR (Drug Therapy[MeSH Subheading])) OR (randomized[Title/Abstract] OR randomised[Title/Abstract] OR placebo[Title/Abstract] OR randomly[Title/Abstract] OR trial[Title/Abstract] OR groups[Title/Abstract])) NOT (animal[mh] NOT humans[mh]) |
| **#6** | #4 and #5  ((((periodontal disease[MeSH Terms]) OR (((gingivitis[Title/Abstract]) OR (periodontitis[Title/Abstract])) OR (gum disease[Title/Abstract]))) AND ((curcumin[MeSH Terms]) OR (((curcumin[Title/Abstract]) OR (curcuma[Title/Abstract])) OR (turmeric[Title/Abstract])))) AND ((chlorhexidine[MeSH Terms]) OR (chlorhexidine[Title/Abstract]))) AND (((((randomized controlled trial[Publication Type]) OR (controlled clinical trial[Publication Type])) OR (Drug Therapy[MeSH Subheading])) OR (randomized[Title/Abstract] OR randomised[Title/Abstract] OR placebo[Title/Abstract] OR randomly[Title/Abstract] OR trial[Title/Abstract] OR groups[Title/Abstract])) NOT (animal[mh] NOT humans[mh])) |
