# Supplementary material for: Effect of curcumin compared to chlorhexidine on clinical variables of periodontal health: A systematic review and meta-analysis of randomized controlled trials
Source: Medicine (Baltimore). 2026 Jul 24;105(30):e49862. doi: 10.1097/MD.0000000000049862 (PMC13406067; doi:10.1097/MD.0000000000049862)
Supplement: Supplementary file 2 [file medi-105-e49862-s002.docx]

**Supplementary Table 2**

The detailed searching strategy followed by the PICO searching tree in EMBASE database. Date of search: January 29, 2025.

| **Searching steps** | **Searching strategy of each step** |
| --- | --- |
| **#1 (population)** | 'periodontal disease'/exp OR 'paraodontopathy' OR 'parodentopathy' OR 'parodontal disease' OR 'parodontium disease' OR 'parodontive tissue disease' OR 'peridontal disease' OR 'peridontal tissue disease' OR 'peridontium disease' OR 'periodontal attachment loss' OR 'periodontal disease' OR 'periodontal diseases' OR 'periodontal infection' OR 'periodontium disease' OR 'periodontopathy' OR 'paradontal disease' OR 'paradontopathy' OR 'gingiva disease'/exp OR 'gingiva disease' OR 'gingival diseases' OR 'periodontitis'/exp OR 'paradontitis' OR 'parodontitis' OR 'peridontitis' OR 'periodontitis' OR 'gingivitis'/exp OR 'chronic gingivitis' OR 'gingiva inflammation' OR 'gingival inflammation' OR 'gingivitis' OR 'chronic periodontitis'/exp OR 'adult periodontitis' OR 'chronic periodontitis' OR 'periodontitis chronica' |
| **#2 (Intervention)** | 'curcumin'/exp OR 'curcumin' OR 'curcumine' OR 'nanocurc' OR 'turmeric yellow' OR 'curcuma'/exp OR 'curcuma' OR 'turmeric'/exp OR 'turmeric' |
| **#3 (Comparison)** | 'chlorhexidine'/exp |
| **#4 (Outcome)** | 'gingival index'/exp OR 'loe and silness gingival index' OR 'loe-silness gingival index' OR 'gingival index' OR 'plaque index'/exp OR 'silness and loe plaque index' OR 'silness-loe plaque index' OR 'dental plaque index' OR 'plaque index' OR 'tooth plaque index' OR 'probing depth'/exp OR 'bleeding index'/exp |
| **#5 (study design)** | 'randomized controlled trial'/exp OR 'controlled trial, randomized' OR 'randomised controlled study' OR 'randomised controlled trial' OR 'randomized controlled study' OR 'randomized controlled trial' OR 'trial, randomized controlled' |
| **#6 (final whole searching tree)** | ('periodontal disease'/exp OR 'paraodontopathy' OR 'parodentopathy' OR 'parodontal disease' OR 'parodontium disease' OR 'parodontive tissue disease' OR 'peridontal disease' OR 'peridontal tissue disease' OR 'peridontium disease' OR 'periodontal attachment loss' OR 'periodontal disease' OR 'periodontal diseases' OR 'periodontal infection' OR 'periodontium disease' OR 'periodontopathy' OR 'paradontal disease' OR 'paradontopathy' OR 'gingiva disease'/exp OR 'gingiva disease' OR 'gingival diseases' OR 'periodontitis'/exp OR 'paradontitis' OR 'parodontitis' OR 'peridontitis' OR 'periodontitis' OR 'gingivitis'/exp OR 'chronic gingivitis' OR 'gingiva inflammation' OR 'gingival inflammation' OR 'gingivitis' OR 'chronic periodontitis'/exp OR 'adult periodontitis' OR 'chronic periodontitis' OR 'periodontitis chronica') AND ('curcumin'/exp OR 'curcumin' OR 'curcumine' OR 'nanocurc' OR 'turmeric yellow' OR 'curcuma'/exp OR 'curcuma' OR 'turmeric'/exp OR 'turmeric') AND 'chlorhexidine'/exp AND ('gingival index'/exp OR 'loe and silness gingival index' OR 'loe-silness gingival index' OR 'gingival index' OR 'plaque index'/exp OR 'silness and loe plaque index' OR 'silness-loe plaque index' OR 'dental plaque index' OR 'plaque index' OR 'tooth plaque index' OR 'probing depth'/exp OR 'bleeding index'/exp) AND ('randomized controlled trial'/exp OR 'controlled trial, randomized' OR 'randomised controlled study' OR 'randomised controlled trial' OR 'randomized controlled study' OR 'randomized controlled trial' OR 'trial, randomized controlled') |
