# Supplementary material for: Effect of curcumin compared to chlorhexidine on clinical variables of periodontal health: A systematic review and meta-analysis of randomized controlled trials
Source: Medicine (Baltimore). 2026 Jul 24;105(30):e49862. doi: 10.1097/MD.0000000000049862 (PMC13406067; doi:10.1097/MD.0000000000049862)
Supplement: Supplementary file 3 [file medi-105-e49862-s003.docx]

**Supplementary Table 3**

The detailed searching strategy by using the search manager in Cochrane Libarary. Date of search: January 29, 2025.

| **Searching steps** | **Searching strategy of each step** |
| --- | --- |
| **#1** | (MeSH descriptor: [Periodontal Diseases] explode all trees) OR ((periodontitis):ti,ab,kw) OR ((gingivitis):ti,ab,kw) OR ((gingival inflammation):ti,ab,kw) OR ((chronic periodontitis):ti,ab,kw) |
| **#2** | (MeSH descriptor: [Curcumin] explode all trees) OR ((curcuma):ti,ab,kw) OR ((turmeric):ti,ab,kw) |
| **#3** | (MeSH descriptor: [Chlorhexidine] explode all trees) OR ((chlorhexidine):ti,ab,kw) |
| **#4** | #1 AND #2 AND #3  ((MeSH descriptor: [Periodontal Diseases] explode all trees) OR ((periodontitis):ti,ab,kw) OR ((gingivitis):ti,ab,kw) OR ((gingival disease):ti,ab,kw) OR ((chronic periodontitis):ti,ab,kw)) AND ((MeSH descriptor: [Curcumin] explode all trees) OR ((curcuma):ti,ab,kw) OR ((turmeric):ti,ab,kw)) AND ((MeSH descriptor: [Chlorhexidine] explode all trees) OR ((chlorhexidine):ti,ab,kw)) |
