# Supplementary material for: Effect of curcumin compared to chlorhexidine on clinical variables of periodontal health: A systematic review and meta-analysis of randomized controlled trials
Source: Medicine (Baltimore). 2026 Jul 24;105(30):e49862. doi: 10.1097/MD.0000000000049862 (PMC13406067; doi:10.1097/MD.0000000000049862)
Supplement: Supplementary file 5 [file medi-105-e49862-s005.docx]

**Supplementary Table 5**

Risk of bias assessment results of the included studies.

| **Study ID** | **Randomization process** | **Timing of identification or recruitment of participants** | **Deviations from intended interventions** | **Mising outcome data** | **Measurement of the outcome** | **Selection of the reported result** | **Overall Bias** |
| --- | --- | --- | --- | --- | --- | --- | --- |
| **Anitha 2015** | Some concerns | Low | Low | Low | Low | Some concerns | Some concerns |
| **Arunachalam 2017** | Some concerns | Low | Low | Low | High | Low | High |
| **Bharathi 2024** | Low | Low | Some concerns | Low | Low | High | High |
| **Chatterjee2017** | Some concerns | Low | Low | Low | High | Low | High |
| **Desai 2020** | Some concerns | Low | Low | Low | High | High | High |
| **Divya2017** | Some concerns | Low | Low | Low | High | Low | High |
| **Gottumukkala2013** | Some concerns | Low | Some concerns | Low | High | Low | High |
| **Gottumukkala2014** | Some concerns | Low | Low | Low | High | Low | High |
| **Guru2020** | Some concerns | Low | Low | Low | High | Low | High |
| **Hugar2016** | Some concerns | Low | High | Low | High | Low | High |
| **Jalaluddin2019** | Some concerns | Low | High | Low | High | Low | High |
| **Jaswal 2014** | Some concerns | Low | High | Low | High | Low | High |
| **Kandwal 2015** | Some concerns | Low | High | Low | High | Low | High |
| **Mali2012** | Some concerns | Low | High | Low | High | Low | High |
| **Mirza 2020** | Some concerns | Low | High | Low | High | Low | High |
| **Muglikar2013** | Some concerns | Low | High | Low | High | Low | High |
| **Paschoal 2015** | Some concerns | Low | Some concerns | High | Low | Low | High |
| **Pulikkotil2015** | Low | Low | Low | High | Low | Low | High |
| **Sarkar 2023** | Low | Low | Low | Low | Low | High | High |
| **Siddharth 2020** | Some concerns | Low | Low | Low | Low | Low | Some concerns |
| **Singh 2015** | Some concerns | Low | High | Low | High | Low | High |
| **Singh 2018** | Low | Low | Some concerns | Low | High | Low | High |
| **Singh2021** | Some concerns | Low | Low | Low | High | High | High |
| **Singhla 2017** | Some concerns | Low | High | Low | High | Low | High |
| **Suhag 2007** | Some concerns | Low | High | Low | High | Low | High |
| **Waghmare 2011** | High | Low | Low | Low | Low | Low | High |
