# Supplementary material for: Effect of curcumin compared to chlorhexidine on clinical variables of periodontal health: A systematic review and meta-analysis of randomized controlled trials
Source: Medicine (Baltimore). 2026 Jul 24;105(30):e49862. doi: 10.1097/MD.0000000000049862 (PMC13406067; doi:10.1097/MD.0000000000049862)
Supplement: Supplementary file 6 [file medi-105-e49862-s006.docx]

**Supplementary Table 6**

The detailed characteristics of included RCTs.

| **Author and published year** | **published time** | **Age of subjects (years) (range, mean ± SD) (range))** | **Gender (female:male)** | **No. of Patients** | **No. of sites** | **sample size  (small or large)** | **Sites of curcumin group** | **Sites of chlorhexidine group** | **Intervention Group** | **Control Group** | **Disease type** | **Follow-up time** | **Outcome evaluation** | **SRP treatment** | **application method** | **application type** |
| --- | --- | --- | --- | --- | --- | --- | --- | --- | --- | --- | --- | --- | --- | --- | --- | --- |
| **Anitha 2015** | **earlier study of 5 years before** | **range: 20-50; mean ± SD: 47±2.7.** | **10:20** | **30** | **60** | **small (n<50)** | **30** | **30** | **1% curcumin gel** | **0.1% chlorhexidine gel** | **periodontits** | **15 and 30 days** | **PD, AL, GI, PI, microbiological parameter.** | **Having received SRP.** | **subgingivally delivered into periodontal pockets** | **topical application** |
| **Arunachalam 2017** | **earlier study of 5 years before** | **range: 25-60** | **18:12** | **30** | **90** | **small (n<50)** | **30** | **30** | **Two intervention groups:  Group 2: 0.2% chlorhexidine mouthwash; Group 3: 0.1% curcumin mouthwash.** | **Group 1: saline.** | **gingivitis** | **4 weeks** | **PI, GI, ROM.** | **Without receiving SRP.** | **mouthwash** | **general fullmouth** |
| **Bharathi 2024** | **recent study within 5 years** | **range: 20-40** | **/** | **81** | **81** | **large (n>50)** | **27** | **27** | **Two intervention groups:**  **Group 1: Triphala;**  **Group 2:** **Curcumin.** | **Group 3: Chlorhexidine.** | **gingivitis** | **7 and 14 days** | **PI, GI, BI.** | **Having received SRP.** | **mouthwash** | **general fullmouth** |
| **Chatterjee2017** | **earlier study of 5 years before** | **range: 20-40** | **/** | **150** | **150** | **large (n>50)** | **50** | **50** | **Two intervention groups:  Group 1: CMN mouthwash (0.3%–5.4% of raw turmeric); Group 3: 0.2% CHX mouthwash.** | **Group 2: Placebo  (2 g of coconut oil, 1 g of mint, 15 g of propylene glycol, tween 60 of 20 g, and distilled water)** | **gingivitis** | **7, 14, and 28 days** | **PI, GI, BI.** | **Having received SRP.** | **mouthwash** | **general fullmouth** |
| **Desai 2020** | **recent study within 5 years** | **range: 18-55** | **2:10** | **12** | **36** | **small (n<50)** | **12** | **12** | **Two intervention groups:**  **Group 1: 2% turmeric gel; Group 2: 1% chlorhexidine gel** | **Group III: placebo gel (500mg Carbopol-934, 10ml ofdeionized water, triethanolamine).** | **periodontits** | **7, 21, and 45 days** | **GI, PI, PD.** | **Having received SRP.** | **delivered into the selected sites using a syringe with a needle attached to it.** | **topical application** |
| **Divya2017** | **earlier study of 5 years before** | **range: ≥15** | **/** | **60** | **60** | **small (n<50)** | **30** | **30** | **0.1% curcumin mouthwash;** | **0.2% CHX mouthwash.** | **gingivitis** | **21 days** | **PI, GI, GBI.** | **Without receiving SRP.** | **mouthwash** | **general fullmouth** |
| **Gottumukkala2013** | **earlier study of 5 years before** | **range: 30-55** | **14:12** | **26** | **46** | **small (n<50)** | **23** | **23** | **Two intervention groups:  1% curcumin solution**； 0.2% chlorhexidine gluconate. | **saline** | **periodontits** | **1, 3 and 6 months** | **BOP, PI, PD, microbiological parameter.** | **Having received SRP.** | **subgingival irrigation** | **topical application** |
| **Gottumukkala2014** | **earlier study of 5 years before** | **range: 25-55** | **/** | **60** | **120** | **large (n>50)** | **60** | **60** | **Curcumin collagen sponge** | **Chlorhexidine chip** | **periodontits** | **1, 3 and 6 months** | **PI, GI, PD, AL, microbiological parameter.** | **Having received SRP.** | **subgingivally delivered into periodontal pockets** | **topical application** |
| **Guru2020** | **recent study within 5 years** | **range: 25-50** | **/** | **45** | **45** | **small (n<50)** | **15** | **15** | **Two intervention groups:  Group 2: 1% chlorhexidine gel; Group 3: 2% curcumin nanogel.** | **without any local drug delivery.** | **periodontits** | **21 and 45 days.** | **PI, GI, PD, AL,  microbiological parameter.** | **Having received SRP.** | **subgingivally delivered into periodontal pockets** | **topical application** |
| **Hugar2016** | **earlier study of 5 years before** | **range: 25–50** | **/** | **30** | **60** | **small (n<50)** | **30** | **30** | **2% curcumin gel** | **0.2% chlorhexidine gel** | **periodontits** | **30 and 45 days** | **PI, GI, BI, PD.** | **Having received SRP.** | **subgingivally delivered into periodontal pockets** | **topical application** |
| **Jalaluddin2019** | **recent study within 5 years** | **range: 25-45** | **/** | **60** | **60** | **small (n<50)** | **20** | **20** | **Two intervention groups: Group 2: 1% Curcuma longa L. extract solution**； Group 3: 0.2% chlorhexidine solution. | **Group 1: without applying any drug.** | **periodontits** | **4 and 8 weeks.** | **PI, GI, PD, antibacterial sensitivity.** | **Having received SRP.** | **subgingival irrigation** | **topical application** |
| **Jaswal 2014** | **earlier study of 5 years before** | **range: 21-55** | **3:12** | **15** | **45** | **small (n<50)** | **15** | **15** | **Two intervention groups: Group 1: 2% curcumin gel**； Group 2: 1% chlorhexidine gel. | **Group 3: without applying any drug.** | **periodontits** | **30 and 45 days** | **PI, GI, PD, AL.** | **Having received SRP.** | **subgingivally delivered into periodontal pockets** | **topical application** |
| **Kandwal 2015** | **earlier study of 5 years before** | **range: >18** | **/** | **60** | **60** | **small (n<50)** | **30** | **30** | **Group A: Curcumin gel (10 mg C.longa extract)** | **Group B: 2% chlorhexidine gel** | **gingivitis** | **14, and 21 days** | **PI, GI.** | **Without receiving SRP.** | **topical application  on gums via vaccupress customized applicator trays** | **topical application** |
| **Mali2012** | **earlier study of 5 years before** | **range: ≥15** | **/** | **60** | **60** | **small (n<50)** | **30** | **30** | **Group A: 0.2% chlorhexidine mouthwash;** | **Group B: 0.1% curcumin mouthwash.** | **gingivitis** | **14, and 21 days** | **PI, GI, microbiological parameter.** | **Without receiving SRP.** | **mouthwash** | **general fullmouth** |
| **Mirza 2020** | **recent study within 5 years** | **range: 30-50** | **17:43** | **60** | **60** | **small (n<50)** | **20** | **20** | **Two intervention groups: Group A: 1% curcumin Group B: 0.12% chlorhexidine** | **without any local drug delivery.** | **periodontits** | **3 and 7 days** | **PI, GI, PD, AL,  total protein (TP), C-reactive protein (CRP), albumin,  and alkaline phosphatase (ALP) enzyme.** | **Having received SRP.** | **subgingival irrigation** | **topical application** |
| **Muglikar2013** | **earlier study of 5 years before** | **range: 20-40;** | **/** | **30** | **30** | **small (n<50)** | **10** | **10** | **Two intervention groups: Group 1: chlorhexidine mouthwash; Group 2: curcumin mouthwash;** | **Group 3: without applying any drug.** | **gingivitis** | **7, 14, and 21 days** | **PI, GI.** | **Having received SRP.** | **mouthwash** | **topical application** |
| **Paschoal 2015** | **earlier study of 5 years before** | **range: 13-18** | **/** | **45** | **45** | **small (n<50)** | **15** | **15** | **Two intervention group: Group I: 2% chlorhexidine varnish; Group III: c-PACT (curcumin at 1.5 mg.mL-1) exposed to blue LED light at 450 nm** | **Group II: placebo varnish** | **gingivitis** | **1 and 3 months** | **PI, GBI.** | **Without receiving SRP.** | **photodynamic antimicrobial chemotherapy** | **topical application** |
| **Pulikkotil2015** | **earlier study of 5 years before** | **range: 18-35. Group I: range: 18-27; mean±SD: 21.95±4.68; Group II: range: 17-29; mean ± SD: 20.60±4.53; Group III: range: 18-36; mean ± SD: 23.25 ± 4.82.** | **/** | **56** | **56** | **small (n<50)** | **19** | **19** | **Three intervention grpups: Group I: 1% curcumin gel**； Group II: 1% chlorhexidine gel; Group III: gel containing 0.25% chlorhexidine and 1% metronidazole. | **Control group was not designed in this study.** | **gingivitis** | **29 and 60 days** | **PI, GI, PD, BOP.** | **Without receiving SRP.** | **self-administered gel  in the tooth shield** | **topical application** |
| **Sarkar 2023** | **recent study within 5 years** | **range:：18-40** | **/** | **20** | **20** | **small (n<50)** | **10** | **10** | **0.2% chlorhexidine gluconate mouthwash** | **turmeric mouthrinse** | **gingivitis** | **2, 3 and 4 weeks** | **OHI-S, PI, GI.** | **Without receiving SRP.** | **mouthwash** | **general fullmouth** |
| **Siddharth 2020** | **recent study within 5 years** | **range: ≥30 years** | **5:20** | **25** | **50** | **small (n<50)** | **25** | **25** | **2% curcumin gel** | **0.2% chlorhexidine gel** | **periodonitis** | **1 and 3 months** | **BI, PD, AL, microbiological parameter.** | **Having received SRP.** | **subgingivally delivered into periodontal pockets** | **topical application** |
| **Singh 2015** | **earlier study of 5 years before** | **range: 20-35** | **/** | **40** | **40** | **small (n<50)** | **20** | **20** | **Group B: curcumin gel** | **Group A: 0.2% chlorhexidine gel** | **gingivitis** | **2 and 3 weeks** | **PI, GI, BI.** | **Without receiving SRP.** | **self-administered gel after brushing** | **topical application** |
| **Singh 2018** | **earlier study of 5 years before** | **range: 30-50** | **18:22** | **40** | **120** | **small (n<50)** | **40** | **40** | **Two intervention groups: Group 1: chlorhexidine gluconate chip;  Group 2: curcumin chip;** | **Group 3: without applying any drug.** | **periodontitis** | **1 and 3 months** | **PI, GI, PD, AL.** | **Having received SRP.** | **subgingivally delivered into periodontal pockets** | **topical application** |
| **Singh2021** | **recent study within 5 years** | **Range: 20-40** | **/** | **81** | **81** | **large (n>50)** | **21** | **21** | **Two intervention group: Group A: 6% triphala mouthwash; Group B: curcumin mouthwash** | **Group C: chlorhexidine mouthwash** | **gingivitis** | **7 and 14 days** | **PI, GI.** | **Having received SRP.** | **mouthwash** | **general fullmouth** |
| **Singhla 2017** | **earlier study of 5 years before** | **range: 21-45** | **/** | **30** | **30** | **small (n<50)** | **10** | **10** | **Group A: 0.1% curcumin;  Group B: meswak (HIORA) irrigation.** | **Group C: 0.2% chlorhexidine irrigation.** | **periodontits** | **21 days** | **PI, GI, PD, AL.** | **Having received SRP.** | **subgingival irrigation** | **topical application** |
| **Suhag 2007** | **earlier study of 5 years before** | **/** | **/** | **20** | **200** | **large (n>50)** | **50** | **50** | **Two intervention groups:  Irrigated sites with 1% curcumin; Irrigated sites with 0.2% chlorhexidine.** | **Two control groups**： Irrigated sites with 0.9% saline; Non-irrigated sites. | **periodontitis** | **15 and 21 days** | **PD, BOP.** | **Having received SRP.** | **subgingival irrigation** | **topical application** |
| **Waghmare 2011** | **earlier study of 5 years before** | **range: 20-35** | **/** | **100** | **100** | **large (n>50)** | **50** | **50** | **Group 2: curcumin mouthwash.** | **Group 1: chlorhexidine mouthwash.** | **gingivitis** | **14 and 21 days** | **PI, GI.** | **Without receiving SRP.** | **mouthwash** | **general fullmouth** |
