# Supplementary material for: Effect of curcumin compared to chlorhexidine on clinical variables of periodontal health: A systematic review and meta-analysis of randomized controlled trials
Source: Medicine (Baltimore). 2026 Jul 24;105(30):e49862. doi: 10.1097/MD.0000000000049862 (PMC13406067; doi:10.1097/MD.0000000000049862)
Supplement: Supplementary file 7 [file medi-105-e49862-s007.docx]

**Supplementary Table 7**

The subgroup analysis of studies, respectively for the five different parameters (i.e., PI, GI, PD, AL, and BI).

| **Covaiates** | **Study subgroup** | **No of studies** | **SMD  (Standard mean difference)** | **lower 95% CI (Conference Interval)** | **upper 95% CI (Conference Interval)** | **weight (%)** | **z value  (Tests of overall subgroup effect size)** | ***p* value (Test of overall subgroup effect size)** | **Heterogeneity- I^2^ (%)** | **Heterogeneity- *p* value** |
| --- | --- | --- | --- | --- | --- | --- | --- | --- | --- | --- |
| **Plaque Index (PI):** | | | | | | | | | | |
| **Overall** |  | 43 | -0.041 | -0.245 | 0.163 | 100 | -0.396 | 0.692 | 83.30% | <0.001 |
| **Published time** | recent studies within 5 years | 14 | -0.358 | -0.791 | 0.075 | 29.19 | -1.620 | 0.105 | 82.30% | <0.001 |
|  | Earlier studies of 5 years before | 29 | 0.083 | -0.151 | 0.318 | 70.81 | 0.696 | 0.486 | 84.10% | <0.001 |
| **Disease type** | gingivitis | 26 | -0.125 | -0.369 | 0.119 | 60.74 | -1.005 | 0.315 | 82.70% | <0.001 |
|  | periodontitis | 17 | 0.088 | -0.269 | 0.446 | 39.26 | 0.485 | 0.628 | 82.90% | <0.001 |
| **SRP treatment** | Without receiving SRP treatment | 16 | -0.194 | -0.505 | 0.118 | 37.44 | -1.219 | 0.223 | 83.80% | <0.001 |
|  | Having received SRP treatment | 27 | 0.049 | -0.211 | 0.308 | 62.56 | 0.368 | 0.713 | 80.90% | <0.001 |
| **Application type** | general fullmouth | 17 | -0.015 | -0241 | 0.212 | 74.68 | -0.126 | 0.900 | 83.20% | <0.001 |
|  | topical application | 26 | -0.133 | -0.619 | 0.352 | 25.32 | -0.538 | 0.591 | 85.00% | <0.001 |
| **Follow-up time** | short term within 1 month | 32 | 0.033 | -0.198 | 0.264 | 70.95 | 0.282 | 0.778 | 81.60% | <0.001 |
|  | long term longer than 1 month | 11 | -0.133 | -0.619 | 0.352 | 29.05 | -0.538 | 0.591 | 85.00% | <0.001 |
| **Age of subjects** | adults | 41 | -0.042 | -0.249 | 0.166 | 96.11 | -0.391 | 0.696 | 80.70% | 0.023 |
|  | adolescents | 2 | -0.032 | -1.422 | 1.359 | 3.89 | -0.044 | 0.965 | 80.70% | 0.023 |
| **Sample size** | < 50 | 34 | 0.025 | -0.204 | 0.253 | 77.16 | 0.210 | 0.833 | 81.50% | <0.001 |
|  | ≥ 50 | 9 | -0.263 | -0.725 | 0.199 | 22.84 | -1.118 | 0.692 | 88.50% | <0.001 |
| **Gingival Index (GI):** | | | | | | | | | | |
| **Overall** |  | 37 | 0.033 | -0.216 | 0.281 | 100 | 0.258 | 0.797 | 87.80% | <0.001 |
| **Published time** | recent studies within 5 years | 13 | -0.269 | -0.873 | 0.335 | 33.65 | -0.873 | 0.383 | 90.40% | <0.001 |
|  | Earlier studies of 5 years before | 24 | 0.169 | -0.098 | 0.436 | 66.35 | 1.241 | 0.215 | 86.50% | <0.001 |
| **Disease type** | gingivitis | 24 | -0.022 | -0.285 | 0.241 | 65.22 | -0.163 | 0.870 | 84.90% | <0.001 |
|  | periodontitis | 13 | 0.124 | -0.442 | 0.689 | 34.78 | 0.429 | 0.668 | 91.00% | <0.001 |
| **SRP treatment** | Without receiving SRP treatment | 14 | 0.071 | -0.234 | 0.375 | 38.64 | 0.455 | 0.649 | 82.90% | <0.001 |
|  | Having received SRP treatment | 23 | -0.013 | -0.394 | 0.368 | 61.36 | -0.065 | 0.948 | 89.60% | <0.001 |
| **Application type** | general fullmouth | 18 | -0.063 | -0.348 | 0.221 | 48.14 | -0.437 | 0.662 | 78.10% | <0.001 |
|  | topical application | 19 | 0.136 | -0.266 | 0.538 | 51.86 | 0.664 | 0.507 | 91.60% | <0.001 |
| **Follow-up time** | short term within 1 month | 29 | -0.026 | -0.269 | 0.218 | 78.87 | -0.207 | 0.836 | 84.80% | <0.001 |
|  | long term longer than 1 month | 8 | 0.233 | -0.619 | 1.085 | 21.13 | 0.535 | 0.593 | 93.00% | <0.001 |
| **Sample size** | < 50 | 28 | 0.102 | -0.195 | 0.399 | 74.34 | 0.371 | 0.502 | 87.60% | <0.001 |
|  | ≥ 50 | 9 | -0.164 | -0.650 | 0.321 | 25.66 | -0.664 | 0.507 | 89.50% | 0.001 |
| **Probing Depth (PD):** | | | | | | | | | | |
| **Overall** |  | 27 | 0.883 | 0.413 | 1.347 | 100 | 3.731 | <0.001 | 93.70% | <0.001 |
| **Published time** | recent studies within 5 years | 9 | 0.330 | -0.203 | 0.863 | 34.14 | 1.214 | 0.225 | 81.80% | <0.001 |
|  | Earlier studies of 5 years before | 28 | 1.244 | 0.612 | 1.875 | 65.86 | 3.857 | <0.001 | 95.30% | <0.001 |
| **Disease type** | gingivitis | 2 | -0.301 | -0.754 | 0.152 | 7.68 | -1.302 | 0.193 | 0.00% | 0.498 |
|  | periodontitis | 25 | 0.993 | 0.500 | 1.486 | 92.32 | 3.949 | <0.001 | 94.00% | <0.001 |
| **SRP treatment** | Without receiving SRP treatment | 2 | -0.301 | -0.754 | 0.152 | 7.68 | -1.302 | 0.193 | 0.00% | 0.498 |
|  | Having received SRP treatment | 25 | 0.993 | 0.500 | 1.486 | 92.32 | 3.949 | <0.001 | 94.00% | <0.001 |
| **Follow-up time** | short term within 1 month | 15 | 1.121 | 0.505 | 1.738 | 55.80 | 3.564 | <0.001 | 93.80% | <0.001 |
|  | long term longer than 1 month | 12 | 0.581 | -0.119 | 1.282 | 44.20 | 1.626 | 0.104 | 93.20% | <0.001 |
| **Sample size** | < 50 | 23 | 1.005 | 0.411 | 1.600 | 83.91 | 3.314 | 0.001 | 94.40% | <0.001 |
|  | ≥ 50 | 4 | 0.728 | 0.329 | 1.126 | 16.09 | 3.579 | <0.001 | 75.00% | 0.007 |
| **Attachment Loss (AL):** | | | | | | | | | | |
| **Overall** |  | 13 | 0.539 | 0.019 | 1.058 | 100 | 2.031 | 0.042 | 90.1% | <0.001 |
| **Published time** | recent studies within 5 years | 5 | 0.453 | -0.255 | 1.160 | 38.23 | 1.254 | 0.210 | 83.00% | <0.001 |
|  | Earlier studies of 5 years before | 8 | 0.598 | -0.140 | 1.336 | 61.77 | 1.589 | 0.112 | 92.80% | <0.001 |
| **Followup time** | short term within 1 month | 8 | 0.797 | 0.047 | 1.546 | 61.36 | 2.083 | 0.037 | 92.30% | <0.001 |
|  | long term longer than 1 month | 5 | 0.130 | -0.501 | 0.762 | 38.64 | 0.405 | 0.686 | 81.40% | <0.001 |
| **Sample size** | < 50 | 12 | 0.577 | -0.017 | 1.171 | 91.64 | 1.904 | 0.057 | 90.70% | <0.001 |
|  | ≥ 50 | 1 | 0.131 | -0.228 | 0.489 | 8.36 | 0.714 | 0.475 | － | － |
| **Bleeding Index (BI):** | | | | | | | | | | |
| **Overall** |  | 14 | -0.044 | -0.208 | 0.295 | 100 | 0.339 | 0.735 | 77.70% | <0.001 |
| **Published time** | recent studies within 5 years | 4 | 0.204 | -0.0069 | 0.477 | 27.20 | 1.461 | 0.144 | 0.00% | 0.697 |
|  | Earlier studies of 5 years before | 10 | -0.011 | -0.318 | 0.297 | 72.80 | -0.068 | 0.946 | 81.70% | <0.001 |
| **Disease type** | gingivitis | 10 | -0.127 | -0.337 | 0.083 | 72.84 | -1.182 | 0.237 | 59.50% | 0.008 |
|  | periodontitis | 4 | 0.396 | -0.304 | 1.096 | 27.16 | 1.109 | 0.267 | 84.70% | <0.001 |
| **SRP treatment** | Without receiving SRP treatment | 5 | -0.366 | -0.587 | -0.144 | 34.95 | -3.236 | 0.001 | 34.30% | 0.193 |
|  | Having received SRP treatment | 9 | 0.196 | -0.106 | 0.498 | 65.05 | 1.274 | 0.203 | 71.50% | <0.001 |
| **Application type** | general fullmouth | 6 | -0.018 | -0.204 | 0.167 | 44.99 | -0.194 | 0.846 | 3.70% | 0.393 |
|  | topical application | 8 | 0.097 | -0.324 | 0.517 | 55.01 | 0.450 | 0.653 | 86.00% | <0.001 |
| **Follow-up time** | short term within 1 month | 12 | 0.038 | -0.240 | 0.316 | 88.15 | -0.267 | 0.789 | 80.70% | <0.001 |
|  | long term longer than 1 month | 2 | 0.074 | -0.360 | 0.507 | 11.85 | 0.334 | 0.738 | 0.00% | 0.579 |
| **Age of subjects** | adults | 12 | 0.023 | -0.246 | 0.293 | 90.53 | 00.170 | 0.865 | 80.50% | <0.001 |
|  | adolescents | 2 | 0.251 | -0.343 | 0.844 | 9.47 | 0.828 | 0.408 | 0.00% | 0.929 |
| **Sample size** | < 50 | 9 | 0.052 | -0.324 | 0.427 | 62.11 | 0.271 | 0.786 | 84.00% | <0.001 |
|  | ≥ 50 | 5 | 0.018 | -0.184 | 0.219 | 37.89 | 0.172 | 0.864 | 6.30% | 0.371 |
