# Supplementary material for: Effect of curcumin compared to chlorhexidine on clinical variables of periodontal health: A systematic review and meta-analysis of randomized controlled trials
Source: Medicine (Baltimore). 2026 Jul 24;105(30):e49862. doi: 10.1097/MD.0000000000049862 (PMC13406067; doi:10.1097/MD.0000000000049862)
Supplement: Supplementary file 8 [file medi-105-e49862-s008.docx]

**Supplementary Table 8**

The meta-regression analysis for the potential moderators between studies, respectively for the five different parameters (PI, GI, PD, AL, and BI).

| **Moderators** | **Coefficient** | **Standard Error (SE)** | **t value** | **P value**  **(significance level)** | **Lower 95% CI**  **(Confidence Interval)** | **Upper 95% CI**  **(Confidence Interval)** |
| --- | --- | --- | --- | --- | --- | --- |
| **Plaque Index (PI):** | | | | | |  |
| Published time (recent studies within 5 years; earlier studies of 5 years before) | 0.5416097 | 0.283573 | 1.91 | 0.064 | -0.0340741 | 1.117294 |
| Disease type (gingivitis/periodontitis) | -0.0583379 | 0.6165146 | -0.09 | 0.925 | -1.309929 | 1.193253 |
| SRP treatment (without receiving; having received) | 0.5961735 | 0.4521981 | 1.32 | 0.196 | -.3218373 | 1.514184 |
| Application type (general fullmouth; topical application) | 0.0448849 | 0.4214414 | 0.11 | 0.916 | -0.8106867 | 0.9004564 |
| Follow-up time (short term within one month; long term longer than 1 month) | -0.3649151 | 0.3335764 | -1.09 | 0.281 | -1.042111 | 0.3122809 |
| Age of subjects (adolescents; adults) | -0.1821587 | 0.6720686 | -0.27 | 0.788 | -1.546531 | 1.182213 |
| Sample size (<50; ≥50) | -0.4233659 | 0.405559 | -1.04 | 0.304 | -1.246695 | 0.3999626 |
| **Gingival Index (GI):** | | | | | |  |
| Published time (recent studies within 5 years; earlier studies of 5 years before) | 0.5149771 | 0.3801804 | 1.35 | 0.186 | -0.2614549 | 1.291409 |
| Disease type (gingivitis; periodontitis) | 0.3001279 | 0.7620606 | 0.39 | 0.696 | -1.256207 | 1.856463 |
| SRP treatment (without receiving; having received) | -0.1579094 | 0.5340042 | -0.30 | 0.769 | -1.248491 | 0.9326726 |
| Application type (general fullmouth; topical application) | -0.0613297 | 0.5630773 | -0.11 | 0.914 | -1.211287 | 1.088627 |
| Follow-up time (short term within one month; long term longer than 1 month) | 0.2357452 | 0.4958712 | 0.48 | 0.638 | -0.7769589 | 1.248449 |
| Sample size (<50; ≥50) | -0.031763 | 0.5341325 | -0.06 | 0.953 | -1.122607 | 1.059081 |
| **Probing Depth (PD)：** | | | | | |  |
| Published time (recent studies within 5 years; earlier studies of 5 years before) | 2.036048 | 1.579796 | 1.29 | 0.211 | -1.240248 | 5.312344 |
| Disease type (gingivitis; periodontitis) | 2.687133 | 2.708252 | 0.99 | 0.332 | -2.929438 | 8.303704 |
| Follow-up time (short term within one month; long term longer than 1 month) | -0.2498969 | 1.406218 | -0.18 | 0.861 | -3.166215 | 2.666422 |
| Sample size (<50; ≥50) | -1.709388 | 2.056274 | -0.83 | 0.415 | -5.973839 | 2.555064 |
| **Attachment Loss (AL):** | | | | | |  |
| Published time (recent studies within 5 years; earlier studies of 5 years before) | 0.0016692 | 0.7171172 | 0.00 | 0.998 | -1.652006 | 1.655345 |
| Follow-up time (short term within one month; long term longer than 1 month) | -.7836104 | 0.7159653 | -1.09 | 0.306 | -2.434629 | 0.8674086 |
| Parameter AL (CAL; RAL; CRAL) | -0.1124139 | 0.5319492 | -0.21 | 0.838 | -1.339091 | 1.114263 |
| Sample size (<50; ≥50) | -0.8334368 | 1.265666 | -0.66 | 0.529 | -3.752067 | 2.085194 |
| **Bleeding Index (BI):** | | | | | |  |
| Published time (recent studies within 5 years; earlier studies of 5 years before) | 0.1296405 | 0.3450861 | 0.38 | 0.718 | -0.6863585 | 0.9456395 |
| Follow-up time (short term within one month; long term longer than 1 month) | -.4113087 | 0.4953708 | -0.83 | 0.434 | -1.582675 | 0.7600571 |
| Disease type (gingivitis/periodontitis) | 0.6984729 | 0. 6692015 | 1.04 | 0.331 | -0.8839372 | 2.280883 |
| Application type (general fullmouth; topical application) | -.2421983 | 0.5657526 | -0.43 | 0.681 | -1.579991 | 1.095594 |
| Age of subjects (adolescents; adults) | -0.950552 | 0.5735595 | -1.66 | 0.141 | -2.306805 | 0.4057007 |
| SRP treatment (without receiving; having received) | 0.3570884 | 0.5397425 | 0.66 | 0.529 | -0.9191999 | 1.633377 |
